# Lithium Use During Pregnancy in 14 Countries

**DOI:** 10.1001/jamanetworkopen.2024.51117

**Published:** 2024-12-16

**Authors:** Felix Wittström, Carolyn E. Cesta, Brian T. Bateman, Marie Bendix, Mette Bliddal, Adrienne Y. L. Chan, Yongtai Cho, Eun-Young Choi, Jacqueline M. Cohen, Sarah Donald, Mika Gissler, Alys Havard, Sonia Hernandez-Diaz, Krista F. Huybrechts, Bianca Kollhorst, Edward Chia-Cheng Lai, Maarit K. Leinonen, Brian M. H. Li, Kenneth K. C. Man, Vanessa W. S. Ng, Lianne Parkin, Laura Pazzagli, Lotte Rasmussen, Ran S. Rotem, Tania Schink, Ju-Young Shin, Duong T. Tran, Ian C. K. Wong, Helga Zoega, Johan Reutfors

**Affiliations:** 1 Centre for Pharmacoepidemiology, Department of Medicine Solna, Karolinska Institutet, Stockholm, Sweden; 2 Department of Anesthesiology, Perioperative and Pain Medicine, Stanford University School of Medicine, Stanford, California; 3 Centre for Psychiatry Research, Department of Clinical Neuroscience, Karolinska Institutet, Stockholm, Sweden; 4 Stockholm Health Care Services, Region Stockholm, Stockholm, Sweden; 5 Department of Clinical Sciences, Umeå University, Umeå, Sweden; 6 Clinical Pharmacology, Pharmacy and Environmental Medicine, Department of Public Health, University of Southern Denmark, Odense; 7 Research Unit OPEN, Department of Clinical Research, University of Southern Denmark, Odense, Denmark; 8 School of Pharmacy, Aston University, Birmingham, United Kingdom; 9 Department of Pharmacology and Pharmacy, Li Ka Shing Faculty of Medicine, University of Hong Kong, Hong Kong; 10 School of Pharmacy, Sungkyunkwan University, Seoul, South Korea; 11 Department of Chronic Diseases and Centre for Fertility and Health, Norwegian Institute of Public Health, Oslo, Norway; 12 Department of Preventive and Social Medicine, Dunedin School of Medicine, University of Otago, New Zealand; 13 Department of Data and Analytics, Finnish Institute for Health and Welfare, Helsinki, Finland; 14 Department of Molecular Medicine and Surgery, Karolinska Institutet, Stockholm, Sweden; 15 Academic Primary Health Care Centre, Region Stockholm, Stockholm, Sweden; 16 National Drug and Alcohol Research Centre, University of New South Wales (UNSW) Sydney, Sydney, Australia; 17 School of Population Health, Faculty of Medicine and Health, UNSW Sydney, Sydney, Australia; 18 Department of Epidemiology, Harvard T.H. Chan School of Public Health, Boston, Massachusetts; 19 Division of Pharmacoepidemiology and Pharmacoeconomics, Department of Medicine, Brigham and Women’s Hospital and Harvard Medical School, Boston, Massachusetts; 20 Department of Biometry and Data Management, Leibniz Institute for Prevention Research and Epidemiology, Bremen, Germany; 21 School of Pharmacy, Institute of Clinical Pharmacy and Pharmaceutical Sciences, College of Medicine, National Cheng Kung University, Tainan, Taiwan; 22 Teratology Information Service, Emergency Medicine and Services, University of Helsinki and Helsinki University Hospital, Helsinki, Finland; 23 Laboratory of Data Discovery for Health (D24H), Hong Kong Science Park, Hong Kong; 24 Research Department of Practice and Policy, UCL (University College London) School of Pharmacy, London, United Kingdom; 25 Centre for Medicines Optimisation Research and Education, University College London Hospitals NHS (National Health Service) Foundation Trust, London, United Kingdom; 26 Clinical Epidemiology Division, Department of Medicine Solna, Karolinska Institutet, Stockholm, Sweden; 27 Maccabitech Institute for Research and Innovation, Maccabi Healthcare Services, Tel Aviv, Israel; 28 Department of Environmental Health, Harvard T.H. Chan School of Public Health, Boston, Massachusetts; 29 Department of Clinical Epidemiology, Leibniz Institute for Prevention Research and Epidemiology, Bremen, Germany; 30 School of Pharmacy, Medical Sciences Division, Macau University of Science and Technology, Macau, China; 31 Centre of Public Health Sciences, Faculty of Medicine, University of Iceland, Reykjavík

## Abstract

**Importance:**

In pregnancy, the benefits of lithium treatment for relapse prevention in psychiatric conditions must be weighed against potential teratogenic effects. Currently, there is a paucity of information on how and when lithium is used by pregnant women.

**Objective:**

To examine lithium use in the perinatal period.

**Design, Setting, and Participants:**

This cohort study used individual-level data of pregnancies from January 1, 2000, to December 31, 2021, in Australia, Denmark, Finland, Germany, Hong Kong, Iceland, Israel, New Zealand, Norway, South Korea, Sweden, Taiwan, the UK, and 2 cohorts in the US. Analyses were performed from September 1 to November 30, 2023.

**Exposures:**

The prevalence of lithium use as the proportion of pregnancies with at least 1 prescription fill or prescription within 3 months before pregnancy until childbirth was estimated using a common protocol. Lithium use during pregnancy by trimester and in the 3 months before and after pregnancy was examined.

**Main Outcomes and Measures:**

Comparison of prevalence between the first and last 3-year periods of available data.

**Results:**

Among 21 659 454 pregnancies from all collaborating sites, the prevalence of lithium use ranged from 0.07 per 1000 pregnancies in Hong Kong to 1.56 per 1000 in the US publicly insured population. Lithium use increased per 1000 pregnancies in 10 populations (Australia [0.60 to 0.74], Denmark [0.09 to 0.51], Finland [0.10 to 0.29], Iceland [0.24 to 0.99], Israel [0.25 to 0.37], Norway [0.24 to 0.47], South Korea [0.30 to 0.44], Sweden [0.42 to 1.07], the UK [0.07 to 0.10], and Taiwan [0.15 to 0.19]), remained stable in 4 populations (Germany [0.17 to 0.16], Hong Kong [0.06 to 0.06], and the publicly [1.50 to 1.34] and commercially [0.38 to 0.36] insured US populations), and decreased in 1 population (New Zealand [0.54 to 0.39]). Use of lithium decreased with each trimester of pregnancy, while prevalence of postpartum use was similar to prepregnancy levels. The proportion of lithium use in the second trimester compared with the prepregnancy period ranged from 2% in South Korea to 80% in Denmark.

**Conclusions and Relevance:**

Prevalence of lithium use in pregnant women over the past 2 decades varied markedly between populations. Patterns of use before, during, and after pregnancy suggest that many women discontinued lithium use during pregnancy and reinitiated treatment after childbirth, with large variations between countries. These findings underscore the need for internationally harmonized guidelines, specifically for psychiatric conditions among pregnant women that may benefit from lithium treatment.

## Key Points

**Question:**

How often is lithium used during pregnancy and has its use in pregnancy changed in the last 2 decades?

**Findings:**

In this cohort study of 21 659 454 pregnancies, the prevalence of lithium use varied substantially among 14 countries, ranging from 0.07 to 1.56 per 1000 pregnancies. Use increased over time in 10 populations, was stable in 4 (including 2 populations in the US), decreased in 1, and was lower in the second and third trimesters compared with the prepregnancy, first trimester, and postpartum periods.

**Meaning:**

These findings suggest that differences in guidelines, clinical practice, and population characteristics may contribute to variations in lithium use during pregnancy.

## Teaser

This cohort study examines the use of lithium during pregnancy over the past 2 decades in 14 countries.

## Introduction

Lithium is the first-line treatment for relapse prevention in bipolar disorder and may also be used in the treatment of other psychiatric conditions, such as recurrent depressive disorder and schizoaffective disorder.^1,2^ Given the severe and chronic nature of these conditions, along with the elevated risk of relapse,^3^ the continuation or the initiation of lithium treatment in pregnancy may be justified. However, the benefits of relapse prevention must be carefully weighed against potential adverse effects on both mother and child.^3^

Only a few studies have examined the prevalence of lithium use in pregnancy. Three studies, 2 from the UK^4,5^ and 1 from the US,^6^ investigated lithium use as a proportion of all pregnancies. Findings indicated that lithium was used in 0.0067% and 0.015% of pregnancies in the UK data sources^4,5^ and 0.1% of pregnancies in the US data source.^6^ Additionally, studies from Hong Kong,^7^ Denmark,^8^ and Australia^9^ found that between 7% and 28% of women with bipolar disorder were prescribed lithium during pregnancy.

The studies from Hong Kong,^7^ Denmark,^8^ and Australia^9^ were based on small study populations or samples from single countries or did not specifically report on use before, during, and after pregnancy. Furthermore, there is a paucity of information on trends in lithium use over time and a lack of information on other psychotropic drugs used by pregnant women treated with lithium. Such in-depth knowledge can inform future studies investigating the benefits and risks associated with lithium treatment during this vulnerable period. Therefore, our objective was to examine the use of lithium in pregnant women throughout the pregnancy period over the past 2 decades in 14 countries, including Australia, Denmark, Finland, Germany, Hong Kong, Iceland, Israel, New Zealand, Norway, South Korea, Sweden, Taiwan, the UK, and the US.

## Methods

### Setting, Design, and Data Sources

We conducted a drug utilization study of 15 cohorts using data from population-based health registers or health care utilization databases between January 1, 2000, and December 31, 2021. Data were combined through a common protocol, ensuring standardized definitions of pregnancy and drug use. All pregnancies resulting in birth (livebirth or stillbirth), having reached between 12 and 22 weeks of gestation, depending on the database, were included except for Israel, South Korea, and the US cohorts, which included pregnancies resulting in livebirths only. Detailed information on years covered, data sources, and population coverage for each database is presented in eTable 1 in Supplement 1. This study was approved by the ethical review boards in each country or institution, as applicable, and each waived the requirement for obtaining informed consent because of the secondary use of existing data. Details on ethical approvals are provided in eTable 2 in Supplement 1. This report followed the Strengthening the Reporting of Observational Studies in Epidemiology (STROBE) reporting guidelines.

For the Nordic countries (Denmark, Finland, Iceland, Norway, and Sweden), we linked information on filled prescriptions and births from the entire population using the civil personal registration number assigned to each resident at birth or upon immigration.^10^ For Australia, pharmaceutical claims data and birth data were probabilistically linked, covering all births in the state of New South Wales.^11^ For Germany, health claims data covering one-quarter of the population were used.^12,13^ For Hong Kong and Taiwan, we used population-wide databases containing prescription and birth information, linked through a unique patient identification number.^14,15^ For Israel, health claims data from the second-largest health maintenance organization covering one-quarter of the population were used.^16^ For New Zealand, we linked databases with nationwide prescription fill and birth data through the National Health Index number.^17^ For South Korea, we used claims data from a nationwide public insurance database.^18^ For the UK, prescription and birth information records from general practitioners were linked in a database covering approximately 10% of the population.^19^ For the US, we included insurance claims data from 2 cohorts, Medicaid Analytic eXtract–Transformed Medicaid Statistical Information System Analytic Files (MAX),^20^ covering nationwide publicly insured individuals, and Merative MarketScan Commercial Claims and Encounters database (MarketScan), covering commercially insured individuals,^21^ with data from almost all states.

### Lithium Use

Lithium use was identified using the Anatomical Therapeutic Chemical Classification System code N05AN01. Alternative coding systems were used for some databases, as described in eTable 1 in Supplement 1. We used prescription fill data in all populations except Taiwan, South Korea, and the UK, for which we used prescribing records. We defined lithium use by identifying at least 1 prescription fill or prescription in the pregnancy period, defined as the interval between 90 days before the first day of the last menstrual period (LMP) until birth. Additionally, we investigated lithium use in each trimester and in the 3 months before and after pregnancy, The prepregnancy period was defined as 90 days before LMP until the day before LMP; first trimester, LMP to 97 days after LMP; second trimester, 98 to 202 days after LMP; third trimester, 203 days after LMP to birth; and the postpartum period, 1 to 90 days after childbirth.

### Other Psychotropic Drug Use

In pregnancies with lithium use in the pregnancy period, we described other psychotropic drug use (≥1 prescription fill or prescription for antiepileptics, antipsychotics, or antidepressants) from 1 year before LMP to birth. Anatomical Therapeutic Chemical Classification System codes used in the study are listed in eTable 3 in Supplement 1.

### Statistical Analysis

Data were analyzed from September 1 to November 30, 2023. We calculated the prevalence of lithium use in the pregnancy period per 1000 pregnancies, overall and by maternal age category (≤24, 25-34, and ≥35 years) in each population. To examine lithium use over time, we calculated population-specific prevalence for each year of childbirth. To account for large fluctuations year to year, we calculated 3-year moving averages of the yearly prevalence of use. We calculated prevalence ratios between the last and first 3-year prevalence averages within each population, with 95% Wald CIs.

Among lithium users in the pregnancy period, we calculated the proportion of pregnancies with other psychotropic drug use. Finally, to describe lithium use before, during, and after pregnancy, we calculated the proportion of pregnancies with lithium use in the 5 distinct periods before pregnancy, during the 3 trimesters, and after pregnancy for each population. To describe the magnitude of the change in lithium use before and after the first trimester when pregnancy recognition occurs, we calculated the proportion of pregnancies with lithium use in the second trimester compared with the prepregnancy period.

Analyses of individual-level data were performed according to a common protocol within each country, and only the summary data were shared with the coordinating center (Sweden). Five databases had restrictions on the sharing of small numbers (<11 in the US MAX, <5 in Denmark and South Korea, and <3 in Taiwan and New Zealand). In such cases, we used half of the restriction limit as a floating-point number ending with 0.5 in the analysis. For all other databases, counts of less than 5 were so reported. Analyses were performed using R, version 2023.09.0 + 463 (R Project for Statistical Computing).^22^

## Results

From the 15 populations in 14 countries, a total of 21 659 454 pregnancies were included, of which 8314 had at least 1 prescription fill for lithium in the pregnancy period. The prevalence of lithium use ranged from 0.07 per 1000 pregnancies in Hong Kong to 1.56 per 1000 pregnancies in US MAX (Table 1). In most of the populations (Australia, Denmark, Finland, Germany, Hong Kong, Iceland, Israel, New Zealand, Norway, Sweden, and the UK), the highest prevalence of lithium use was in the oldest maternal age category (≥35 years), whereas in South Korea and US MarketScan, the highest prevalence was in the youngest maternal age category (≤24 years).

**Table 1.** Prevalence of Lithium Use in Pregnancy by Population and Maternal Age

Population (years included) by maternal age	No. of pregnant women	No. of lithium users^a^	Prevalence per 1000 pregnancies	Prevalence per 1000 pregnancies in last/first 3 y of available data	Prevalence ratio (95% CI) per 1000 pregnancies
Australia (2014-2019)	566 933	381	0.67		
≤24 y	58 917	45	0.76	0.74/0.60	1.23 (0.86-1.74)
25-34 y	368 260	221	0.60		
≥35 y	139 748	115	0.82		
Denmark (2000-2021)	1 348 203	374	0.28		
≤24 y	158 011	24	0.15	0.51/0.09	5.98 (2.42-14.78)
25-34 y	928 153	216	0.23		
≥35 y	262 039	134	0.51		
Finland (2005-2016)	701 172	144	0.21		
≤24 y	121 243	16	0.13	0.29/0.10	2.98 (1.14-7.76)
25-34 y	444 295	92	0.21		
≥35 y	135 634	36	0.27		
Germany (2004-2020)	1 852 666	316	0.17		
≤24 y	175 027	21	0.12	0.16/0.17	0.92 (0.44-1.95)
25-34 y	1 196 058	156	0.13		
≥35 y	481 581	139	0.29		
Hong Kong (2001-2018)	526 302	36	0.07		
≤24 y	39 055	<5	<0.13	0.06/0.06	0.98 (0.06-14.87)
25-34 y	314 812	16	0.05		
≥35 y	172 434	18	0.10		
Iceland (2004-2017)	61 316	48	0.78		
≤24 y	12 276	9	0.73	0.99/0.24	4.11 (0.46-36.79)
25-34 y	37 589	25	0.66		
≥35 y	11 451	14	1.22		
Israel (2000-2021)	829 933	248	0.30		
≤24 y	99 450	22	0.22	0.37/0.25	1.46 (0.63-3.40)
25-34 y	502 233	127	0.25		
≥35 y	228 250	99	0.43		
New Zealand (2006-2020)	865 701	421	0.49		
≤24 y	195 443	65	0.33	0.39/0.54	0.72 (0.41-1.26)
25-34 y	484 491	217	0.45		
≥35 y	185 767	139	0.75		
Norway (2005-2020)	940 193	340	0.36		
≤24 y	134 027	43	0.32	0.47/0.24	1.97 (1.03-3.78)
25-34 y	618 519	201	0.32		
≥35 y	187 647	96	0.51		
South Korea (2010-2021)	4 574 294	1469	0.32		
≤24 y	279 604	173	0.62	0.44/0.30	1.48 (1.16-1.89)
25-34 y	3 334 012	927	0.28		
≥35 y	960 678	369	0.38		
Sweden (2006-2019)	1 505 389	1126	0.75		
≤24 y	201 467	103	0.51	1.07/0.42	2.49 (1.73-3.59)
25-34 y	970 425	678	0.70		
≥35 y	333 495	345	1.03		
Taiwan (2010-2020)^b^	2 120 040	353	0.17		
≤24 y	1 338 079	35	0.03	0.19/0.15	1.26 (0.76-2.08)
25-34 y	183 938	191	1.04		
≥35 y	591 377	127	0.21		
UK (2000-2021)^c^	3 165 384	254	0.08		
≤24 y	109 350	<5	<0.05	0.10/0.07	1.32 (0.45-3.89)
25-34 y	966 175	122	0.13		
≥35 y	387 516	118	0.30		
US MAX (2000-2018)	1 583 037	2467	1.56		
≤24 y	795 832	1020	1.28	1.34/1.50	0.90 (0.67-1.20)
25-34 y	653 453	1205	1.84		
≥35 y	133 752	242	1.81		
US MarketScan (2003-2020)	1 018 891	337	0.33		
≤24 y	30 165	19	0.63	0.36/0.38	0.96 (0.34-2.73)
25-34 y	667 218	189	0.28		
≥35 y	321 508	129	0.40		

Abbreviations: MAX, Medicaid Analytic eXtract–Transformed Medicaid Statistical Information System Analytic Files.

^a^ Indicates at least 1 prescription fill 90 days before last menstrual period until birth.

^b^ Missing maternal age information for 6646 pregnancies with lithium use.

^c^ Missing maternal age information for 12 pregnancies with lithium use.

**Figure 1.**
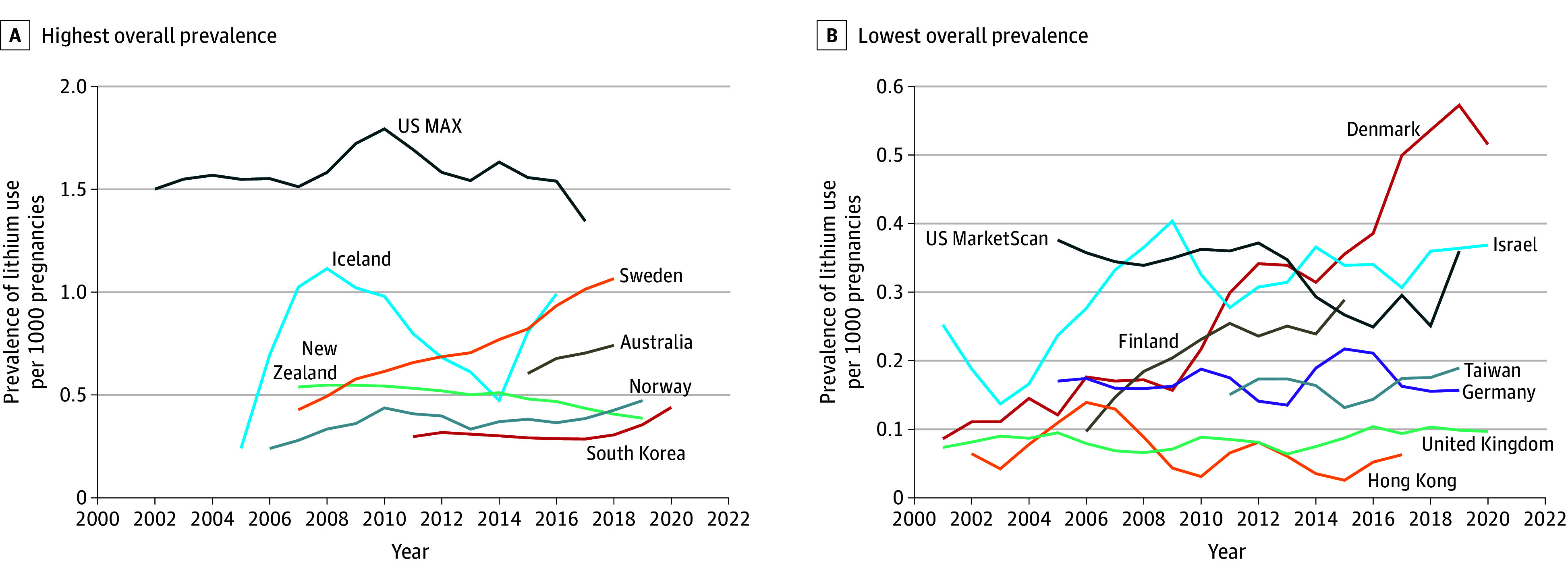
Prevalence of Lithium Use Over Time Lithium use in the pregnancy period is defined as at least 1 prescription fill from 90 days before the last menstrual period until birth. Prevalence of use is shown as a 3-year moving average. MAX indicates Medicaid Analytic eXtract–Transformed Medicaid Statistical Information System Analytic Files.

When comparing the averages of the last and first 3-year periods of available data (Table 1), prevalence of lithium use increased in 10 populations (Australia [0.60 to 0.74], Denmark [0.09 to 0.51], Finland [0.10 to 0.29], Iceland [0.24 to 0.99], Israel [0.25 to 0.37], Norway [0.24 to 0.47], South Korea [0.30 to 0.44], Sweden [0.42 to 1.07], the UK [0.07 to 0.10], and Taiwan [0.15 to 0.19]), remained stable in 4 populations with a prevalence ratio between 0.90 and 1.10 (Germany [0.17 to 0.16], Hong Kong [0.06 to 0.06], US MAX [1.50 to 1.34], and US MarketScan [0.38 to 0.36]), and decreased in 1 population (New Zealand [0.54 to 0.39]). The largest increase in lithium use in pregnancy over time was in the Nordic countries, where the prevalence ratio ranged between 1.97 (95% CI, 1.03-3.78) and 5.98 (95% CI, 2.42-14.78). The 3-year moving average of the yearly prevalence of lithium use in the pregnancy period for all years is depicted in Figure 1.

Table 2 shows the proportion of pregnancies with lithium use in the pregnancy period with use of other psychotropic drugs. The median proportion of pregnancies with antiepileptic use was 32%; with antipsychotic use, 55%; and with antidepressant use, 56%. Specifically, the proportion of pregnancies with use of antiepileptics ranged from 13 of 254 (5%) in the UK to 230 of 353 (65%) in Taiwan; use of antipsychotics, from 7 of 254 (3%) in the UK to 1167 of 1469 (79%) in South Korea; and use of antidepressants, from 83 of 248 (33%) in Israel to 1845 of 2467 (75%) in US MAX.

**Table 2.** Other Psychotropic Drug Use Among Women Using Lithium in Pregnancy

Population	Lithium use, No. of pregnancies^a^	No. (%) of pregnancies with other drug use^b^		
		Antiepileptics	Antipsychotics	Antidepressants
Australia	381	49 (13)	194 (51)	154 (40)
Denmark	374	113 (30)	196 (52)	187 (50)
Finland	144	49 (34)	97 (67)	66 (46)
Germany	316	39 (12)	161 (51)	191 (60)
Hong Kong	36	14 (39)	25 (69)	14 (39)
Iceland	48	12 (25)	27 (56)	34 (71)
Israel	248	79 (32)	130 (52)	83 (33)
New Zealand	421	136 (32)	306 (73)	236 (56)
Norway	340	109 (32)	189 (56)	141 (41)
South Korea	1469	646 (44)	1167 (79)	1042 (71)
Sweden	1126	403 (36)	556 (49)	602 (53)
Taiwan	353	230 (65)	268 (76)	221 (63)
UK	254	13 (5)	7 (3)	145 (57)
US MAX	2467	1233 (50)	1563 (63)	1845 (75)
US MarketScan	337	197 (58)	172 (51)	237 (70)

Abbreviation: MAX, Medicaid Analytic eXtract–Transformed Medicaid Statistical Information System Analytic Files.

^a^ Indicates at least 1 prescription fill 90 days before last menstrual period until birth.

^b^ Indicates at least 1 prescription fill for each respective drug class from 1 year before last menstrual period to birth.

**Figure 2.**
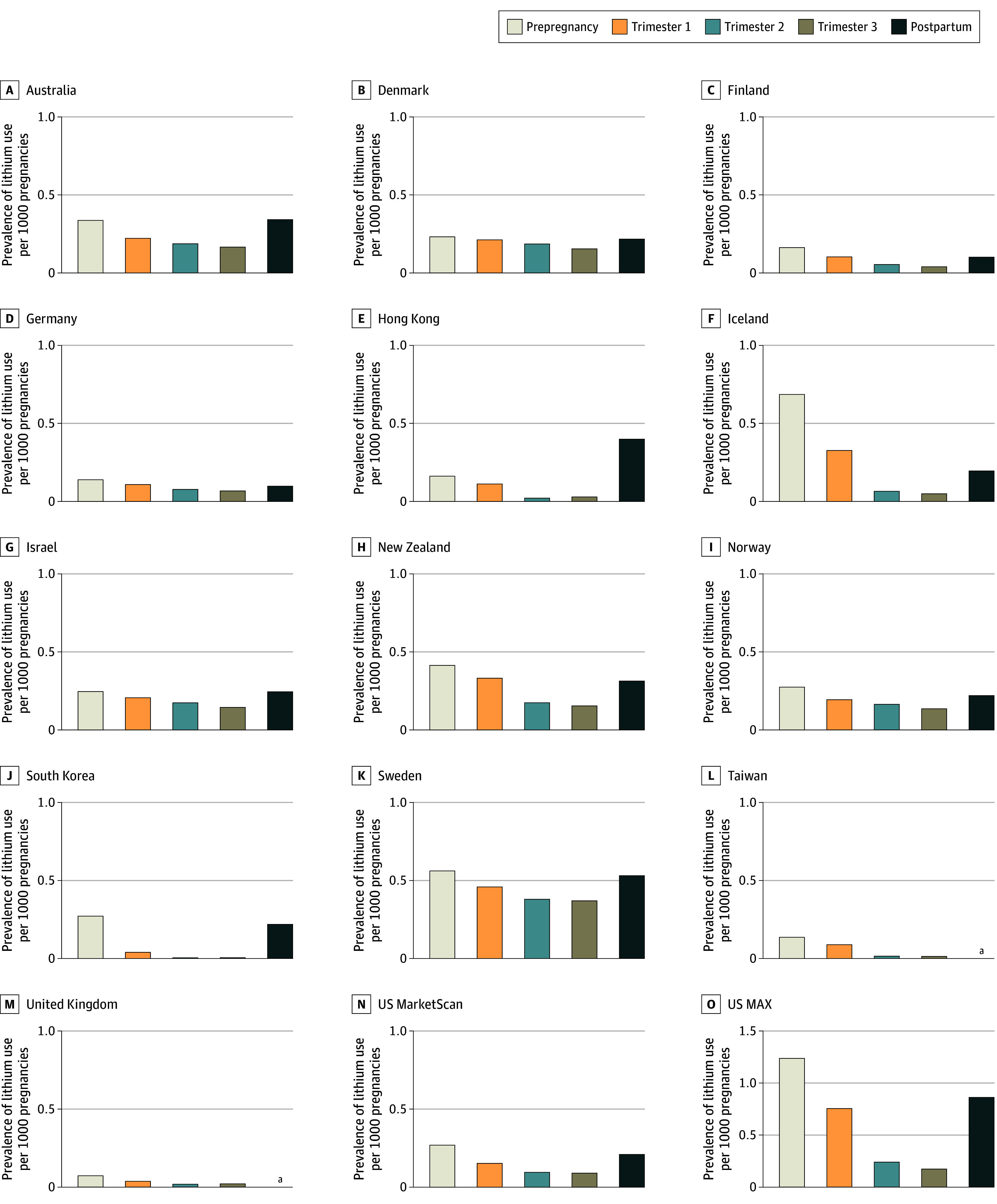
Prevalence of Lithium Use Before, During, and After Pregnancy The scales on the y-axes differ (panel O [US MAX; Medicaid Analytic eXtract–Transformed Medicaid Statistical Information System Analytic Files] ranges from 0 to 1.5) and are not directly comparable to the other panels. Prepregnancy indicates up to 90 days before the last menstrual period (LMP); first trimester, up to 97 days after LMP; second trimester, between 98 and 202 days after LMP; third trimester, more than 203 days after LMP until birth; and postpartum, up to 90 days after birth. ^a^Data on lithium use for the postpartum period were not available.

Figure 2 shows the prevalence of lithium use before, during, and after pregnancy for each database. In all populations, we observed a lower prevalence of lithium use in all 3 trimesters of pregnancy compared with the prepregnancy period, with an increase in the prevalence of use in the postpartum period. This pattern was most notable in South Korea while being least evident in Denmark: the proportion of pregnancies with lithium use in the second trimester compared with the prepregnancy period was 2% in South Korea and 80% Denmark. eTable 4 in Supplement 1 reports the number of pregnancies with lithium use for each pregnancy period, and the relative use in the second trimester compared with the prepregnancy period.

## Discussion

In this cohort study including over 21 million pregnancies across 14 countries during the most recent 2 decades, the use of lithium among pregnant women displayed substantial variation between populations. The prevalence of use over time was observed to increase in 10 populations, remain stable in 4, and decrease in 1. Lithium use was lower in the second and third trimesters, compared with the prepregnancy and postpartum levels.

The prevalence of lithium use in the previous single-database studies aligns with the lowest and highest prevalences of lithium use found in the present study. Our estimated prevalence of lithium use in the UK falls between the previous estimates reported in UK populations (0.06 and 0.15 per 1000 pregnancies).^4,5^ The prevalence of lithium use in the US MAX population of 1.56 per 1000 pregnancies in the present study is somewhat higher than that reported from a Tennessee Medicaid population (1.0 per 1000 pregnancies).^6^ This is likely attributable to the present study considering prescriptions filled during the 90 days before LMP, whereas the earlier study only considered prescriptions filled during the 30 days before LMP with a 1-day supply overlapping with the first trimester. A generally low prevalence of lithium use was also found previously in Hong Kong,^7^ using similar data to the present study, where lithium was the least frequently dispensed mood stabilizer in pregnancy in women with bipolar disorder.

Contrary to prior population-based studies that reported a decrease in lithium use in pregnant women over time for almost all populations,^6,7,8^ we observed an increase or stable prevalence of use over the study period. A contributing factor may be our inclusion of more recent years of data. While the increase in prevalence of use was persistent throughout the years of available data for most countries in the present study, studies published between 2017 and 2020 highlighted that the risk for congenital malformations in lithium users was lower than previously reported, potentially changing the risk-benefit considerations for pregnant women and their clinicians.^23,24,25,26^ For nonpregnant patients with bipolar disorder, use of lithium has been decreasing over the past 2 decades in 6 of the populations in the present study (Denmark, Finland, Germany, Hong Kong, Taiwan, the UK, and the US).^27,28,29,30,31,32^ It is therefore notable that we see a stable or increased use of lithium in pregnant women in these specific countries.

Of the previous longitudinal studies investigating lithium use across the pregnancy period,^5,7,8^ all found lithium use to decrease as the pregnancy progressed, in agreement with our results. In a previous study from the UK,^5^ only 33% of women prescribed lithium 3 months before the start of pregnancy received further prescriptions after the sixth week of pregnancy, suggesting high levels of lithium therapy discontinuation. This pattern of use is also seen for other mood stabilizers and antipsychotics, with a decrease of use in pregnancy compared with the period before pregnancy.^33,34^ For some of our populations (eg, South Korea) there was nearly no use of lithium in the later trimesters, potentially indicating high levels of treatment discontinuation. In other populations, the decrease in use as pregnancy progressed was less pronounced (eg, Denmark), suggesting that a larger proportion continued their treatment.

Differences in lithium use between populations may be explained by varying clinical practices reflecting different regional and national guidelines and values in the risk-benefit analysis. Highlighted in a review of guidelines for the management of bipolar disorder during the perinatal period, there are large differences between several national guidelines, which may lead to suboptimal treatment.^35^ In Australia, Denmark, New Zealand, Sweden, and the US, populations with high lithium use in our study, guidelines highlight the importance of lithium in relapse prevention in pregnancy.^36,37,38,39^ In contrast, in South Korea, where use of lithium was moderate, and in Taiwan, where it was low, treatment guidelines for bipolar disorder suggest it as a third-line treatment or do not mention lithium use in pregnancy,^40,41^ which may place larger responsibility on the individual clinician. Such overarching factors governing clinical practices may also contribute to the marked differences in trends over time, and differences in lithium use patterns across the pregnancy period, observed in the present study. Furthermore, the same clinical guidelines that highlight the importance of lithium relapse prevention in pregnancy also recommend fetal echocardiography in the second trimester to screen for potential cardiac malformations,^36,37,38,39^ which may influence the willingness of mothers to use lithium in pregnancy.

Another influence on the observed differences in lithium use are socioeconomic factors. Within the US, there was a notably higher prevalence of lithium use in the publicly insured cohort (MAX), where mothers are younger and have low economic resources and more psychiatric disorders compared with the commercially insured (MarketScan) cohort, where there may be more pregnancy planners who discontinue use of lithium before pregnancy.^42^ Furthermore, differences in care settings may influence the observed differences in lithium use. Our findings of lower prevalence of lithium use in the UK and very low prevalence of other psychotropic drug use are likely due to the data originating from primary care, which does not include prescriptions by specialists in psychiatry.

One possible explanation for the observed patterns of lithium use across pregnancy is the teratogenic risk associated with fetal exposure to lithium. While the infrastructure for monitoring of fetal malformations may vary between countries, and the true risk of malformations is still unknown, a meta-analysis of 29 studies^23^ found that when comparing lithium-exposed with unexposed pregnancies, the odds ratio for any congenital anomaly was 1.81 (95% CI, 1.35-2.41) and for cardiac anomaly was 1.86 (95% CI, 1.16-2.96). At the same time, continuing lithium use may be warranted due to the high rate of relapse associated with severe psychiatric conditions in the perinatal period, where a review points out that 40% to 70% of pregnant women with bipolar disorder experience a relapse.^3^ The large differences in patterns of use across the pregnancy period observed between our populations likely reflect inconsistent clinical recommendations, putting responsibility for the decision to discontinue or continue to use lithium on mothers and the clinicians treating them.^43^

In most populations, lithium use after childbirth returned to levels similar to use in the prepregnancy period. Although our study was not designed to follow up the treatment trajectories of individual patients, and users in the postpartum period may include women with acute treatment needs (eg, postpartum psychosis), our results suggest that lithium use is often discontinued during pregnancy and reinitiated after childbirth. Most clinical guidelines discourage breastfeeding in women using lithium.^24,37,38^ Our data do not include information on breastfeeding, but the increase in the number of women using lithium after childbirth highlights the need for information on how well postpartum lithium guidelines are adhered to, as well as consequences of lithium exposure through breast milk.

Lithium is the first-line treatment for relapse prevention in bipolar disorder.^1^ In a meta-analysis including data from 8 countries,^44^ the pooled prevalence of bipolar disorder in the perinatal period was 2.6% (95% CI, 1.2%-4.5%). While the only overlap between the 8 countries and the present study was the US, this prevalence of bipolar disorder was far higher than our observed prevalence of lithium use, suggesting that a much smaller fraction of pregnant women with bipolar disorder may be receiving lithium treatment than the number who have the disorder.

### Strengths and Limitations

A strength of this study is the utilization of a common protocol to combine information on lithium use in pregnancy from different databases, allowing for comparison between populations. While differences in population coverage, socioeconomic factors, and what level of care the data cover may still limit comparability, our results also highlight the importance of considering such factors when studying lithium use in pregnancy.

This study also has some limitations. In prescription and health care utilization data, there is no participant recall or reporting bias on medication use during pregnancy. However, data on prescription fills and prescriptions do not capture actual use and adherence to treatment. Differences in prescribing practices between countries may limit uniform interpretation of trends and patterns in lithium use. For example, prescriptions typically cover 30 days of supply in the US, while in the Nordic countries they typically cover 90 days.

## Conclusions

In this cohort study, the prevalence of lithium use during pregnancy over the past 2 decades was found to increase in 10 populations, to be stable in 4, and to decrease in 1. The observed patterns of use across the pregnancy period suggest that many women discontinued lithium use during pregnancy and reinitiated their treatment after childbirth, with large variations between countries. Differences in guidelines, clinical practice, and population characteristics may partially explain the observed variation. Our findings underscore the need for development of internationally harmonized guidelines, specifically for psychiatric conditions in pregnant women that may benefit from lithium treatment.

## Notes

Supplement 1. **eTable 1.** Characteristics of Databases Included in the Study **eTable 2.** Ethical Approval Details for Countries in the Study **eTable 3.** Code Book for Other Psychotropic Drug Use **eTable 4.** Number of Pregnancies With Prescription Fills for Lithium in 5 Distinct Periods Before, During, and After Pregnancy

Supplement 2.
Data Sharing Statement
