# Supplementary material for: Lithium Use During Pregnancy in 14 Countries
Source: JAMA Netw Open. 2024 Dec 16;7(12):e2451117. doi: 10.1001/jamanetworkopen.2024.51117 (PMC11650410; doi:10.1001/jamanetworkopen.2024.51117)
Supplement: Supplement 1. — eTable 1. Characteristics of Databases Included in the Study eTable 2. Ethical Approval Details for Countries in the Study eTable 3. Code Book for Other Psychotropic Drug Use eTable 4. Number of Pregnancies With Prescription Fills for Lithium in 5 Distinct Periods Before, During, and After Pregnancy [file jamanetwopen-e2451117-s001.pdf]

Supplementary Online Content

Wittström F, Cesta CE, Bateman BT, et al. Lithium use during pregnancy in 14 countries. *JAMA Netw Open*. 2024;7(12):e2451117. doi:10.1001/jamanetworkopen.2024.51117

eTable 1. Characteristics of Databases Included in the Study

eTable 2. Ethical Approval Details for Countries in the Study

eTable 3. Code Book for Other Psychotropic Drug Use

eTable 4. Number of Pregnancies With Prescription Fills for Lithium in 5 Distinct Periods Before, During, and After Pregnancy

This supplementary material has been provided by the authors to give readers additional information about their work.

© 2024 Wittström F et al. *JAMA Network Open*.

eTable 1. Characteristics of Databases Included in the Study

**Database (years covered)**	**Data sources**	**Inclusion of outpatient/in-patient, reimbursed/ non-reimbursed drug dispensing data**	**Population covered in the study (including cohort selection criteria, whether stillbirths are included, etc.)**
**Australia** 2014-2019	a) NSW Perinatal Data Collection<br>b) Pharmaceutical Benefits Scheme data (national pharmaceutical dispensing claims data).<br>c) NSW Admitted Patient Data Collection	Subsidized prescription drugs dispensed in outpatient settings and private hospitals. Drugs dispensed during admissions in public hospitals not captured. Dispensations are recorded with modified ATC codes.	Pregnancies resulting in birth (livebirth, or stillbirth) in the state of New South Wales of at least 20 weeks of gestation or at least 400 grams birthweight.
**Denmark** 2000-2021	a) Medical Birth Register<br>b) National Prescription Register<br>c) National Patient Registry<br>d) Civil Registration System	All filled prescriptions at a community pharmacy.	Pregnancies resulting in a livebirth, or stillbirth from gestational week 22.
**Finland** 2005-2016	a) Medical Birth Register<br>b) Register of Reimbursed Drug Purchases<br>c) Care Register for Health Care (Patient Register)	Reimbursed drugs in outpatient care.	Pregnancies resulting in a live birth or a stillbirth from gestation week 22.
**Germany** 2004–2015	German Pharmacoepidemiological Research Database (GePaRD), a healthcare claims database	Dispensed, reimbursed prescription drugs in outpatient care (general practitioners and specialist care).	Pregnancies resulting in live birth or stillbirth (>500 grams) in publicly insured women.
**Hong Kong** 2001–2018	Clinical Data Analysis and Reporting System (CDARS)	Dispensed prescription drugs in public in-and outpatient care.	Pregnancies in public hospitals resulting in live birth or stillbirth.
**Iceland** 2004-2017	a) Medical Birth Register<br>b) National medicines registry<br>b) Prescription Medicines Register<br>c) Patient Register	Dispensed prescription drugs to total population in outpatient care. Hospital administered drugs not available.	Pregnancies resulting in the birth of a live-born infant, and stillbirths from gestational week 22.
**Israel** 2000-2021	Maccabi Healthcare Services (MHS), Israel's second largest integrated health fund.	All prescription medications dispensed at more than 700-affiliated pharmacies	Pregnancies resulting in livebirth in women continuously enrolled in their health plan from at least 1 year before conception through delivery.
**New Zealand** 2006-2020	a) New Zealand Pregnancy Cohort (derived from national Ministry of Health databases: National Maternity Collection, National Minimum Dataset (hospitalisations), Mortality Collection, Laboratory Claims Collection)<br>b) Pharmaceutical Collection<br>c) National Health Index data	Subsidized dispensings of prescription drugs from community pharmacies (hospital administered drugs not included). Dispensations are recorded with Chemical IDs rather than ATC codes.	Pregnancies resulting in the delivery of live or stillborn infants ≥20 weeks of gestation.
**Norway** 2005-2020	a) Medical Birth Register<br>b) Norwegian Prescription Database<br>c) National Patient Register	Prescriptions dispensed to patients outside institutions (community pharmacies).	Pregnancies resulting in live birth or stillbirth from gestation week 12 (at least 22 weeks or 500 grams).

© 2024 Wittström F et al. *JAMA Network Open*.

**South Korea** 2010-2021	Health Insurance Review and Assessment Service (HIRA) claims data, a nationwide public insurance database	All prescribed drugs identified by domestic drug chemical codes.	Pregnancies resulting in livebirth.
**Sweden** 2006-2019	a) Medical Birth Register<br>b) Prescribed Drug Register<br>c) National Patient Register	Dispensed prescription drugs. Hospital administered drugs not included.	Pregnancies resulting in the birth of a live-born infant or stillbirths from gestation week 22.
**Taiwan** 2010-2020	Taiwan's National Health Insurance Research Database (NHIRD)	All prescribed drugs.	Pregnancies resulting in birth (livebirth, or stillbirth) of at least 20 weeks of gestation or at least 500 grams birthweight.
**UK** 2001-2020	Clinical Practice Research Datalink (CPRD) GOLD (Primary care database) Publicly insured	Drugs prescribed in general practice.	Pregnancy-related records are extracted from clinical records of all female patients between the ages of 11 and 49 years. Additional data are extracted from linked babies' records in the first year of life. Only live birth is included.
**US MarketScan** 2003–2020	Merative MarketScan® Commercial Claims and Encounters (MarketScan) database (Healthcare claims database) Commercially insured	Dispensed, reimbursed prescription drugs in outpatient care. Dispensations are identified with generic drug names.	Pregnancies resulting in livebirth in women continuously enrolled in their health plan from 1 year before pregnancy until 3 months after delivery.
**US MAX** 2000–2018	Medicaid Analytic eXtract/ Transformed Medicaid Statistical Information System Analytic Files (MAX) database (Healthcare claims database) Publicly insured	Dispensed, reimbursed prescription drugs in outpatient care. Dispensations are identified with generic drug names.	Pregnancies resulting in livebirth in women continuously enrolled in a state Medicaid program from before 1 year before pregnancy until 3 months after delivery.

© 2024 Wittström F et al. *JAMA Network Open*.

eTable 2. Ethical Approval Details for Countries in the Study

**Country**	**Ethical Board**	**Approval Number / Details**
**Australia**	New South Wales Population and Health Services Research Human Research Ethics Committee<br>Australian Institute of Health and Welfare Ethics Committee	2019/ETH11830, 2023UMB0606<br>EC 2020/2/1130
**Denmark**	Not required by Danish law	Registered at the University of Southern Denmark's inventory (record 11.627)
**Finland**	Not required by Finnish law	THL (Finnish Institute for Health and Welfare)/1673/5.05.00/2019, Kela 117/522/2019
**Germany**	According to the Ethics Committee of the University of Bremen studies based on GePaRD are exempt from institutional review board review.	In Germany, the utilisation of health insurance data for scientific research is regulated by the Code of Social Law. All involved health insurance providers as well as the German Federal Office for Social Security and the Senator for Health, Women and Consumer Protection in Bremen as their responsible authorities approved the use of GePaRD data for this study.
**Hong Kong**	Institutional Review Board of the University of Hong Kong/Hospital Authority Hong Kong West Cluster	UW 20-051
**Iceland**	National Bioethics Committee	VSN-18-123
**Israel**	Maccabi Healthcare Services Helsinki Committee	MHS 0027-19
**New Zealand**	University of Otago Human Ethics Committee (Health)	HD23/037
**Norway**	Regional Committee for Medical and Health Research Ethics	2017/2546/REK sør-øst A
**South Korea**	Institutional Review Board of Sungkyunkwan University	SKKU 2023-11-061
**Sweden**	Swedish Ethical Review Authority (Etikprövningsmyndigheten)	DNR 2015/1826–31/2, 2017/2238–32, 2018/1790–32, 2018/2211–32, 2022/04004–02
**Taiwan**	Chang Gung Medical Foundation Institutional Review Board	202400296B0
**United Kingdom**	The Clinical Practice Research Datalink Research Data Governance Committee	23_003033
**United States**	Mass General Brigham Institutional Review Board	2017P002780

© 2024 Wittström F et al. *JAMA Network Open*.

eTable 3. Code Book for Other Psychotropic Drug Use

Other psychotropic drug use	ATC code* (≥1 prescription fill from 1 year before LMP until birth)
Antiepileptics	N03A
Antipsychotics (excluding lithium)	N05A (excluding N05AN01)
Antidepressants	N06A

* Alternative coding systems were used for some databases and are noted in eTable 1.
ATC: Anatomical Therapeutic Chemical Classification System. LMP: Last menstrual period.

eTable 4. Number of Pregnancies With Prescription Fills for Lithium in 5 Distinct Periods Before, During, and After Pregnancy

Population	PRE	T1	T2 (% of PRE use)	T3	POST
Australia	191	126	106 (55%)	94	194
Denmark	312	286	250 (80%)	208	293
Finland	114	72	38 (33%)	28	71
Germany	257	200	143 (56%)	124	181
Hong Kong	85	59	11 (13%)	15	210
Iceland	42	20	<5 (<12%)	<5	12
Israel	204	171	144 (71%)	120	203
New Zealand	358	287	151 (42%)	133	271
Norway	258	182	154 (60%)	127	207
South Korea	1243	182	21 (2%)	23	1003
Sweden	845	691	572 (68%)	557	800
Taiwan	289	188	32 (11%)	29	Not available
UK	229	119	58 (25%)	67	Not available
US MAX	1,956	1,194	379 (19%)	274	1363
US MarketScan	274	155	97 (35%)	91	213

PRE, up to 90 days before last menstrual period (LMP). T1, first trimester, up to 97 days after LMP. T2, second trimester, between 98 and 202 days after LMP. T3, third trimester, more than 203 days after LMP until birth. POST, up to 90 days after birth.

© 2024 Wittström F et al. *JAMA Network Open*.
