# Supplementary material for: Lithium Use During Pregnancy in 14 Countries
Source: JAMA Netw Open. 2024 Dec 16;7(12):e2451117. doi: 10.1001/jamanetworkopen.2024.51117 (PMC11650410; doi:10.1001/jamanetworkopen.2024.51117)
Supplement: Supplement 2. — Data Sharing Statement [file jamanetwopen-e2451117-s002.pdf]

Data Sharing Statement

Wittström. Lithium Use During Pregnancy in 14 Countries. *JAMA Netw Open*. Published December 16, 2024. doi:10.1001/jamanetworkopen.2024.51117

Data
Data available: No

Additional Information
Explanation for why data not available: Due to data privacy laws, the data cannot be made publicly available.
